# Small Interference RNA Targeting Connexin-43 Improves Motor Function and Limits Astrogliosis After Juvenile Traumatic Brain Injury

**DOI:** 10.1177/1759091419847090

**Published:** 2019-06-13

**Authors:** Aleksandra Ichkova, Andrew M. Fukuda, Nina Nishiyama, Germaine Paris, Andre Obenaus, Jerome Badaut

**Affiliations:** 1 CNRS UMR5287, University of Bordeaux, France; 2 Department of Physiology, Loma Linda University, CA, USA; 3 Department of Pediatrics, Loma Linda University Medical Center, CA, USA; 4 Department of Psychiatry and Human Behavior, Alpert Medical School of Brown University, Providence, RI, USA; 5 Center for Glial-Neuronal Interactions, Division of Biomedical Sciences, University of California, Riverside, CA, USA; 6 Department of Pediatrics, University of California, Irvine, CA, USA

**Keywords:** traumatic brain injury, astrocyte network, gliovascular unit, blood–brain barrier, drug targets

## Abstract

Juvenile traumatic brain injury (jTBI) is the leading cause of death and disability for children and adolescents worldwide, but there are no pharmacological treatments available. Aquaporin 4 (AQP4), an astrocytic perivascular protein, is increased after jTBI, and inhibition of its expression with small interference RNA mitigates edema formation and reduces the number of reactive astrocytes after jTBI. Due to the physical proximity of AQP4 and gap junctions, coregulation of AQP4 and connexin 43 (Cx43) expressions, and the possibility of water diffusion via gap junctions, we decided to address the potential role of astrocytic gap junctions in jTBI pathophysiology. We evaluated the role of Cx43 in the spread of the secondary injuries via the astrocyte network, such as edema formation associated with blood–brain barrier dysfunctions, astrogliosis, and behavioral outcome. We observed that Cx43 was altered after jTBI with increased expression in the perilesional cortex and in the hippocampus at several days post injury. In a second set of experiments, cortical injection of small interference RNA against Cx43 decreased Cx43 protein expression, improved motor function recovery, and decreased astrogliosis but did not result in differences in edema formation as measured via T2-weighted imaging or diffusion-weighted imaging at 1 day or 3 days. Based on our findings, we can speculate that while decreasing Cx43 has beneficial roles, it likely does not contribute to the spread of edema early after jTBI.

## Introduction

In the United States, the annual incidence of nonmilitary-related traumatic brain injury (TBI) is approximately 1.7 million, of which 327,000 are hospitalized and 52,000 die (Faul et al., 2010). Juvenile TBI (jTBI), which is the leading cause of death and disability in children and adolescents, is an important concern because the population group most affected (emergency department visit, hospitalization, and death) are those younger than 5 years, followed by teenagers aged 15 to 19 years old (Faul et al., 2010). The consequences of jTBI are divided into primary and secondary injuries. The initial primary injury results from the direct and immediate biomechanical disruption of the brain tissue. The secondary injuries are part of the development of pathophysiology with delayed molecular mechanisms occurring at sites directly surrounding the impacted site and expanding toward regions remote from the initial impact site (Pop and Badaut, 2011; Plesnila, 2016). The primary injury can only be lessened by taking preemptive cautions, such as wearing helmets, so the goal of potential therapeutics is to minimize the damage caused by the secondary injuries (Morales et al., 2005). The major landmarks of the secondary injury cascade are blood–brain barrier (BBB) disruption and edema with cellular swelling (Pop and Badaut, 2011; Fukuda et al., 2012, 2013). Notably, cerebral edema remains the most significant predictor of poor outcome after injury and accounts for half of the morbidity and mortality after jTBI (Donkin and Vink, 2010; Fukuda et al., 2012, 2013). However, the molecular and cellular mechanisms in edema spread are not yet well understood, and there are no pharmacological treatments available (Clement et al., 2018).

**Figure 1.**
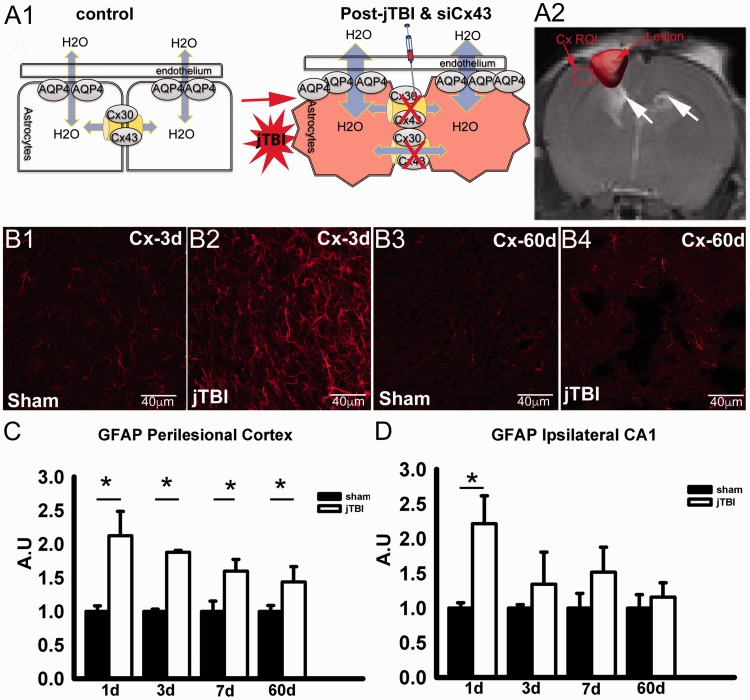
(A) Schematic representation of the working hypothesis and the site of siCx43 injection (A1) gap junctions composed of Cx43 and Cx30 are involved in the spread of edema after TBI. siCx43 inhibits overexpression of Cx43 and limits the edema spread. (A2) Red arrow indicates the siCx43 injection site in the perilesional cortex, and white arrows point ventricle changes. (B) Representative GFAP immunofluorescence confocal images in the perilesional cortex at 3 days (B1, B2) and 60 days after injury (B3, B4), scale bar = 40 μm. (C) GFAP staining quantification in the perilesional cortex showed increased GFAP immunoreactivity at 1 day, 3 days, 7 days, and 60 days after jTBI when compared with sham. (D) GFAP immunoreactivity in the ipsilateral hippocampus (CA1) showed a significant increase only at 1 day. Scale bar = 40 μm (*p < .05). AQP4 = aquaporin 4; Cx43 = connexin 43; Cx30 = connexin 30; GFAP = glial fibrillary acidic protein; jTBI = juvenile traumatic brain injury; ROI = region of interest; siCx43 = small interference RNA against Cx43.

Although various cell types such as neurons, oligodendrocytes, and endothelial cells swell after injury, astrocytes are the first cell types to respond to injury. Astrocytes are known to swell before, and longer than the other cells (Badaut et al., 2011). In fact, perivascular astrocyte endfeet can react within minutes after injury (Grange-Messent et al., 1996; Risher et al., 2009), and this swelling may spread from the primary injury site to distant sites, thus being responsible for the secondary injury cascade. To have a better understanding of this process, examination of astroglial pathophysiology after jTBI is required to successfully target these injury cascades, including edema. The expression of aquaporin 4 (AQP4), a water channel present predominantly on the astrocytes endfeet, has been shown to first decrease and then increase at 3 days and 7 days after jTBI which coincides with both the peak of edema formation at 3 days and edema resolution at 7 days (Fukuda et al., 2012). Early downregulation of AQP4 protein levels using small interference RNA against AQP4 (siAQP4) decreased edema development after jTBI with behavioral improvements both during the acute and chronic phases of injury (Fukuda et al., 2013), suggesting an important role for astrocytic AQP4 in water movements during edema. Importantly, astrocytes are organized in a network where they communicate through specialized channels called gap junctions (Giaume and McCarthy, 1996). The astrocytic network has been proposed to be involved in various brain homeostatic functions, in particular siphoning the excess of potassium ions (K^+^) at distance of the site of activation of neurons (Niermann et al., 2001). Very interestingly, the astrocytic AQP4 has been also proposed to be involved in the K^+^ clearance (Binder et al., 2004, 2006). In addition to the physical relationship between water channels and gap junctions, connexin 43 (Cx43) expression has been linked to the level of expression of AQP4 via coregulation by miRNA (Jullienne et al., 2018). It is very likely that the extent of the edema in the brain tissue during the development of secondary injury after jTBI involves gap junctions (Figure 1(A)).

Astrocytic gap junctions form connections between neighboring astrocytes, allowing the flow of various molecules and water, and the proteins that constitute these gap junctions are the connexins (Giaume et al., 2010). Among the connexins, connexin 43 (Cx43) and connexin 30 (Cx30) are predominantly expressed in astrocytes (Rash et al., 2001; Giaume et al., 2010). Six connexin proteins form a connexon, which is a hemichannel, and when a hemichannel from one cell attaches with another hemichannel of an adjacent cell, a gap junction is established. Therefore, 12 connexins constitute one gap junction (Giaume et al., 2010). Connexin hemichannels have an aqueous pore that selectively permits flow of small endogenous molecules such as second messengers, amino acids, nucleotides, small peptides, and also water (Wallraff et al., 2006; Giaume et al., 2010; Herve and Derangeon, 2013). The hypothesized spread of detrimental factors and toxic metabolites such as sodium and calcium ions, apoptotic factors, lysophospholipids, cyclic adenosine monophosphate, and inositol triphosphate from the primary injury site to more distant sites mediated by gap junctions is referred to as “*bystander effect*” (Andrade-Rozental et al., paper 2000; Perez Velazquez et al., 2003). Several studies have shown increased astrocytic connexin expression in affected brain regions after acute injuries such as stroke, TBI, spinal cord injury, and retinal injury (Chew et al., 2010). However, no studies have characterized the levels of the connexins forming astroglial gap junctions after jTBI and their association with edema spread and astrogliosis.

## Materials and Methods

### General Experimental Setup

For this study, two independent sets of rats were used: in the first set of animals, the changes in the astrocytic network connexin proteins (Cx43, Cx30) and the astrocytic activation marker glial fibrillary acidic protein (GFAP) levels were measured over time after jTBI; in the second set, we evaluated the effect of small interference RNA against Cx43 (siCx43) injection after jTBI on astrocytic markers (Cx43, GFAP, AQP4), neuronal death (with neuronal nuclei [NeuN] marker), and BBB permeability (with immunoglobulin G [IgG]). Edema was assessed using magnetic resonance imaging (MRI), and functional outcome was evaluated with behavior/motor tests.

### Animal Care

All animal care and experiments were conducted according to the Guidelines for Care and Use of Experimental Animals approved by Loma Linda University. All protocols and procedures were in compliance with the U.S. Department of Health and Human Services Guide and were approved by the Institutional Animal Care and Use Committee of Loma Linda University. Postnatal 17-day-old Sprague Dawley male rat pups were housed in a temperature-controlled (22°C–25°C) animal facility on a 12-hr light/dark cycle with standard lab chow and water ad libitum.

### siRNA Preparation

An *in vivo* Cx43 silencing protocol was adapted from previous studies (Badaut et al., 2011). Briefly, SMART-pool® containing 4 siRNA-duplexes against Cx43 (400 ng, siCx34, Dharmacon Research, Horizon Discovery, Cambridge, United Kingdom) and nontargeted siRNA (siGLO RISC-free-control-siRNA, Dharmacon Research, Horizon Discovery, Cambridge, United Kingdom) were mixed with INTERFERin® (Polyplus-transfection, Illkirch, France) diluted in a saline solution (0.9%) containing 5% glucose for a final volume of 5 μL and incubated on ice for 20 minutes before injection. 

### Controlled Cortical Impact and siRNA Injection

Controlled cortical impact (CCI) was carried out on postnatal 17-day-old rat pups as previously described (Ajao et al., 2012; Fukuda et al., 2012, 2013). Rats were anesthetized with isoflurane and placed in a stereotaxic apparatus (David Kopf Instrument, Tujunga, CA, USA). A 5 mm diameter craniotomy over the right hemisphere 3 mm posterior from bregma and 4 mm lateral to midline was performed. Animals were subjected to CCI using an electromagnetic impactor with a 2.7 mm round tip set to impact with a velocity of 6 m/s and a depth of 1.5 mm below the cortical surface (Leica, Richmond, IL, USA). Sham animals received the craniotomy, but without the cortical impact. The craniotomy did not cause damage to the dura mater, which was intact in both the jTBI and sham groups. After CCI, none of the animals had major bleeding or cortical tissue herniation.

siRNA administration was performed as previously described (Fukuda et al., 2013). Injection of siRNA was performed 10 minutes after the injury lateral to the site of the impact using a 30-gauge needle on a Hamilton syringe (3 mm posterior to bregma, 6 mm lateral to midline, and 1.0 mm below cortical surface). The syringe was attached to a nanoinjector (Leica Microsystems, Wetzlar, Germany), and 4 μL of either siCx43 or siGLO was administered at a rate of 0.5 μL/min. After suturing, all pups were placed on a warm heating pad for recovery before being returned to their dams. A second siRNA injection was repeated 2 days after the CCI in all pups that received siRNA using the same injection protocol.

### Magnetic Resonance Imaging

MRI was performed at 1 day and 3 days after jTBI to monitor the process of edema formation and to observe water content and water mobility at the peak of edema in this model (Fukuda et al., 2012, 2013). Pups were lightly anesthetized using isoflurane (1.0%) and imaged on a Bruker Avance 11.7 T (Bruker Biospin, Billerica, MA, USA; Fukuda et al., 2013). Two imaging data sets were acquired: (a) a 10 echo T2-weighted (T2WI) and (b) a diffusion-weighted imaging (DWI) sequence in which each sequence collected 20 coronal slices (1 mm thickness and interleaved by 1 mm). The 11.7T T2WI sequence had the following parameters: TR/TE = 2357.9/10.2 ms, matrix = 128 × 128, field of view = 2 cm, and 2 averages. The DWI sequence had the following parameters: TR/TE = 1096.5/50 ms, two *b* values (116.96, 1044.42 s/mm^2^), matrix = 128 × 128, field of view = 2 cm, and 2 averages.

### Region of Interest and Volumetric Analysis

T2 relaxation values (ms) and apparent diffusion coefficient (ADC) values were quantified using previously published standard protocols (Badaut et al., 2011). Regions of interest (ROIs) were placed on the imaging section with the maximally detected injury using Cheshire (Parexel International Corp., Waltham, MA, USA). Perilesional cortex and ipsilateral hippocampus were delineated on T2WI images and overlaid onto corresponding T2 and ADC maps (Figure 1(A2)). The mean, standard deviation, and area for each ROI were extracted.

**Figure 2.**
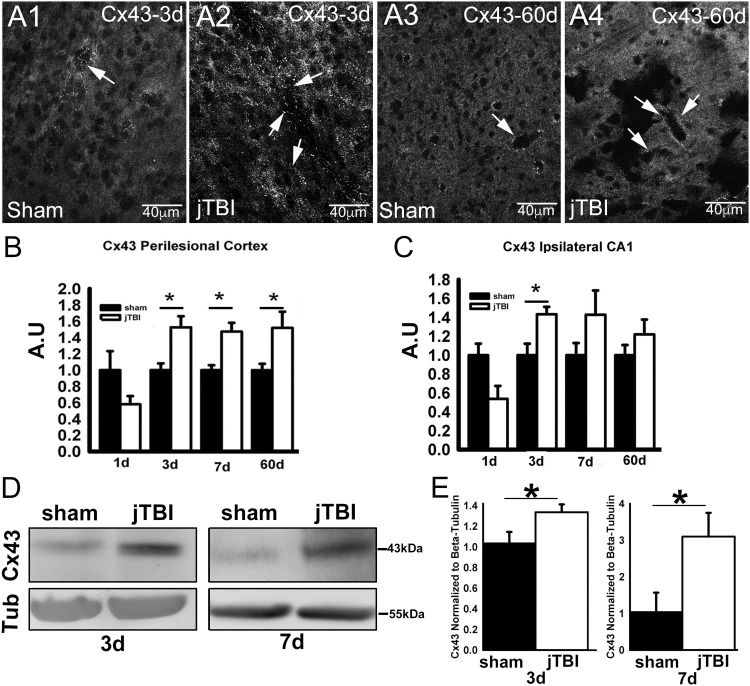
(A) Representative Cx43 immunofluorescence confocal images in the perilesional cortex at 3 days (A1, A2) and 60 days after injury (A3, A4), scale bar = 40 μm. (B) Cx43 staining quantification in the perilesional cortex showed significantly increased Cx43 immunoreactivity at 3 days, 7 days, and 60 days after jTBI when compared with shams. (C) Cx43 immunoreactivity in the ipsilateral hippocampus (CA1) showed a significant increase at 3 days (*p < .05). (D) Western blot of Cx43 at 3 days and 7 days shows a distinct band of Cx43 at 43kDa. (E) Western blot quantification shows a significantly higher expression of Cx43 in the perilesional cortex at both 3 days and 7 days in jTBI compared with shams. Scale bar = 40 μm (*p < .05). jTBI = juvenile traumatic brain injury; Cx43 = connexin 43; kDa = kilo Dalton.

### Behavioral Testing

Foot-fault and rotarod testing was performed at 1 day and 3 days after injury in both the siCx43 and control (siGLO) group. The foot-fault test evaluated sensorimotor coordination and proprioception while the rotarod test tested sensorimotor coordination and balance as previously reported (Ajao et al., 2012). All tests at each time point were carried out on siGLO- and siCx43-treated rats within a 3-hour morning time-block (8–11 a.m.). siGLO- and siCx43-treated rats were interleaved in the testing sequence. To further control for potential confounds, the same tests were administered in the same order at all of the time points, by the same investigators blinded to the experimental groups.

Foot-fault testing was carried out on an elevated platform (50 cm × 155 cm, ClosetMaid, Ocala, FL, USA) with parallel wire bars 1.5 cm apart and raised 100 cm above the floor. Rats were placed in the middle of the platform to freely roam around. When a rodent’s paw (fore- or hindlimb) slipped completely through the wire mesh, it was considered as an individual fault. The average foot-fault score was calculated from the total number of faults from two 60 seconds trials.

Rotarod evaluation (SD Instruments, San Diego, CA, USA) was performed with a rotating 7-cm wide spindle with continuous speed (20 rpm) to evaluate performance during two trials per speed. Latency to fall was the outcome measure of motor coordination and balance. The maximum time spent on the test was 60 seconds, if the rat did not fall, at which point the rotation was halted and the rat was taken off of the spindle. The average time spent on the rotarod from the two trials was calculated and expressed in total time (seconds) for two trials.

### Immunohistochemistry and Image Analysis

For the immunohistochemistry experiments, the animals were transcardially perfused at the respective time point with 4% paraformaldehyde after which brains were extracted and put in 30% sucrose for 48 hours, then stored in –22°C. Coronal sections were cut at 40 μm thickness at –22°C on a cryostat (Leica Biosystems, Wetzlar, Germany) and then mounted on slides for subsequent immunohistochemical analysis (Hirt et al., 2009; Badaut et al., 2011).

The primary antibodies used for immunohistochemistry were rabbit polyclonal antibody for Cx43 (1:100, Abcam, Cambridge, MA, USA; RRID:AB_297976), rabbit polyclonal antibody for Cx30 (1:100, Abcam; RRID:AB_10862289), chicken polyclonal antibody against GFAP (1:1000, Millipore, Billerica, MA, USA; RRID:AB_177521), rabbit polyclonal antibody against AQP4 (1:200, Sigma-Aldrich, St. Louis, MO, USA; RRID:AB_1844967), and rabbit polyclonal against NeuN (1:500, Abcam; RRID:AB_2744676). The secondary antibodies used were InfraRed Dye (IRDye) 800-conjugated affinity-purified donkey-anti-rabbit IgG (1:1000, Rockland, Gilbertsville, PA, USA; RRID:AB_1057615), IRDye 680-conjugated affinity-purified goat-anti-chicken IgG (1:1000, Rockland; RRID:AB_10705059), Alexa 594-conjugated affinity-purified goat anti-rabbit IgG (1:1000, Invitrogen, Carlsbad, CA, USA; RRID:AB_2556545), Alexa 488-conjugated affinity-purified goat anti-rabbit IgG (1:1000, Invitrogen; RRID:AB_2556544), Alexa 568-conjugated affinity-purified goat anti-chicken IgG (1:1000, Invitrogen; RRID:AB_2534098), and Alexa 488-conjugated affinity-purified goat anti-chicken IgG (1:1000, Invitrogen; RRID:AB_2534096).

For immunohistochemistry, sections were washed with phosphate-buffered saline (PBS), blocked with 1% bovine serum albumin (BSA) in PBS, incubated with the respective primary antibodies in PBS containing 0.1% Triton X-100 and 1% BSA overnight, then incubated for 2 hours at room temperature with affinity-purified secondary antibodies conjugated to the desired wavelength in PBS containing 0.1% Triton X-100 and 1% BSA. After washing, brain sections were scanned on an infrared (IR) scanner (Odyssey, Lincoln, NE, USA) to quantify fluorescence for the different ROIs as previously described (Badaut et al., 2011) or imaged under a confocal laser microscope (Zeiss, Oberkochen, Germany) or epifluorescence microscope (Leica Microsystems, Wetzlar, Germany). For sections that were imaged under epifluorescence or confocal microscopy, sections on glass slides were coverslipped with antifading medium VectaShield containing 4’,6-diamidino-2-phenylindole (Vector, Vector Laboratories, Burlingame, CA, USA).

All image acquisition parameters for the same proteins were kept constant for all of the animals for analysis and visualization purposes, and all analyses were carried out in a nonbiased, blinded manner. Analysis of Cx43, GFAP, and NeuN used previously described methods (Fukuda et al., 2013). The slides with the previously mentioned primary antibodies with the secondary antibodies conjugated to the IR wavelength (680 or 800 nm) were scanned on an IR scanner (Odyssey), and images were saved with a resolution of 21 μm per pixel. Identical circular ROIs were drawn in the perilesional cortex and in the ipsilateral CA1 at three different bregma levels (–1.40 mm, –2.56 mm, and –3.80 mm): the bregma level where lesion area was largest, one slice anterior and one posterior. The average fluorescence of these ROIs was calculated to show immunoreactivity of each of the protein and between groups. 4 consecutive images with 20× magnification were taken in the ipsilateral cortex around the lesion site, at 2 different bregma levels (2.56 mm and –3.80 mm). The average fluorescence intensity and the area of staining were measured after processing the images with the Frangi vesselness plugin followed by the Renuy entropy filter in ImageJ (Bethesda, MD, USA) to isolate the vascular staining from the background. Negative control staining where the primary antibody or secondary antibody was omitted showed no detectable labeling.

For IgG extravasation immunohistochemistry, sections were washed with PBS, blocked with 1% BSA in PBS, then incubated for 2 hours at room temperature with IRDye 800-conjugated affinity-purified goat-anti-rat IgG (1:500, Rockland; RRID:AB_1961673) in PBS containing 0.1% Triton X-100 and 1% BSA. After washing, sections were scanned on an IR scanner (Odyssey) to measure the area of extravasation divided by the total brain area per ROI.

### Western Blot

At 3 days and 7 days, brains were freshly extracted from another set of sham and jTBI animals, and the cortical tissue adjacent to the site of impact was collected and frozen for Western blot analysis as previously published (Fukuda et al., 2012). Tissues were placed in a tube with radioimmunoprecipitation assay buffer with protease inhibitor cocktail (Roche, Basel, Switzerland) and sonicated for 30 seconds and stored at –20°C. These samples were then analyzed for total protein concentration by bicinchoninic acid assay (Pierce Biotechnology Inc., Rockford, IL, USA). 10 µg of protein were then subjected to sodium dodecyl sulfate-polyacrylamide gel electrophoresis on a 4% to 12% gel (NuPAGE, Invitrogen). After the electrophoresis, proteins were transferred to a polyvinylidene fluoride membrane (PerkinElmer, Germany). The membrane was incubated with a rabbit polyclonal antibody against Cx43 (1:1000, Abcam; RRID:AB_297976) and a monoclonal antibody against tubulin (1:25,000, Sigma, Switzerland; RRID:AB_477579) in Odyssey blocking buffer (LI-COR, Bioscience, Germany) for 2 hours at room temperature. After washing in PBS for 3 × 10 min, the membrane was incubated with two fluorescence-coupled secondary antibodies (1:10,000, anti-rabbit Alexa-Fluor-680 nm, Molecular Probes, Eugene, OR, USA; RRID:AB_2535736 and anti-mouse IRDye-800-nm, Roche, Germany; RRID:AB_621842) for 2 hours at room temperature. After 3 × 10 minutes washes in PBS, the degree of fluorescence was measured using an IR scanner (Odyssey, LI-COR, Germany) as previously published (Fukuda et al., 2012). The fluorescence value for Cx43 was normalized to tubulin and compared between jTBI and sham at each time point.

### Statistics

One-way analysis of variance (ANOVA) was used for the immunohistochemistry and Western blot analysis to compare the means between the sham and jTBI group at each time point. One-way ANOVA was used for immunohistochemistry analysis to compare the mean between siGLO and siCx43 group as well. Two-way repeated measures ANOVA with a post hoc Bonferroni test was used for the behavior and MRI data. A *p* value less than .05 was considered to be statistically significant. All data in the article are presented as mean ± standard error of the mean. For the first set of experiments, the number of animals was *n* = 24 for sham and *n* = 24 for jTBI animals. For the second set of experiments, the number of animals for all tests was *n* = 5 for the siGLO-treated group and *n* = 7 for siCx43-treated rats.

## Results

### Chronic Astrogliosis After jTBI

The extent of astrogliosis after jTBI was studied via GFAP immunoreactivity, a protein found in the astrocyte cytoskeleton of activated astrocytes (Sofroniew and Vinters, 2010; Figure 1(B)). GFAP immunofluorescence intensity was significantly increased in the perilesional cortex (Figure 1(B2) and (B4)) in the jTBI group compared with the age-matched control group at 1 day, 3 days, 7 days, and 60 days after injury (Figure 1(C)). Unlike the perilesional cortex in which significant increases in astrogliosis were observed at all time points, the ipsilateral hippocampus only had a significant increase (*p* < .05) at 1 day (Figure 1(D)).

### Chronic Increase of Cx43 Expression Associated With Astrogliosis After jTBI

Cx43 staining was observed around the blood vessels with a dotted pattern (Figure 2(A1) and (A3)) in the sham groups at 3 days and 60 days. Cx43 staining was increased in the jTBI rats around blood vessels and within brain parenchyma (Figure 2(A2) and (A4)). Quantification of Cx43 changes at 1 day, 3 days, 7 days, and 60 days post-jTBI using the same ROI used for GFAP immunoreactivity quantification was undertaken. At 1 day, there was no significant difference in the mean immunofluorescence values between the groups (Figure 2(B)), although a 40% decrease in Cx43 immunofluorescence was observed after jTBI (*p* < .125). However, during the edema formation period, which lasts from 3 days to 7 days after injury in this model (Fukuda et al., 2012), there was significant increase in the Cx43 immunofluorescence levels in the perilesional cortex by 53% at 3 days and 47% at 7 days in the jTBI group compared with age-matched sham-operated animals (Figure 2(B), *p* < .05). Furthermore, the increase of the Cx43 immunoreactivity was still observed at 60 days post-injury (Figure 2(B), *p* < .05). Similar increases at 3 days were observed (*p* < .05) in the ipsilateral hippocampus located directly under the site of the impact, when compared with age-matched sham-operated animals (Figure 2(C)). This early Cx43 increase was not sustained after 7 days and 60 days in the hippocampus. No differences were observed between groups in the contralateral cortex and hippocampus (data not shown).

Immunohistochemical quantification was confirmed by Western blot analysis at 3 days and 7 days (Figure 2(D)). The Cx43 Western blot showed a higher intensity in the jTBI group compared with sham animals at 43 kDa. The intensity of Cx43 staining was normalized to the β-tubulin, which was used as a housekeeping protein (Figure 2(D) and (E)). Quantification revealed a significant increase, with ∼30% increase at 3 days and 200% increase at 7 days (Figure 2(E)).

Even though the focus of the study was on Cx43, to assess the overall changes in astrocytic connexins, Cx30 protein changes were also evaluated using IR immunohistochemistry at 1 day, 3 days, 7 days, and 60 days post-jTBI. At 1 day and 3 days, a significant increase was observed in Cx30 immunoreactivity in the perilesional cortex in the injured animals compared with age-matched sham-operated control animals (Supplementary Figure 1(A) and (B), *p* < .05). However, no significant difference between the groups was observed in the perilesional cortex at 7 days and 60 days (Supplementary Figure 1(B)). In the ipsilateral hippocampus located under the site of the impact, a significant increase in Cx30 immunoreactivity was observed at 1 day (*p* < .05) when compared with age-matched sham-operated animals but not at the other time points (Supplementary Figure 1(C)).

### siCx43 Injection Reduces Cx43 Expression Acutely After Injury

**Figure 3.**
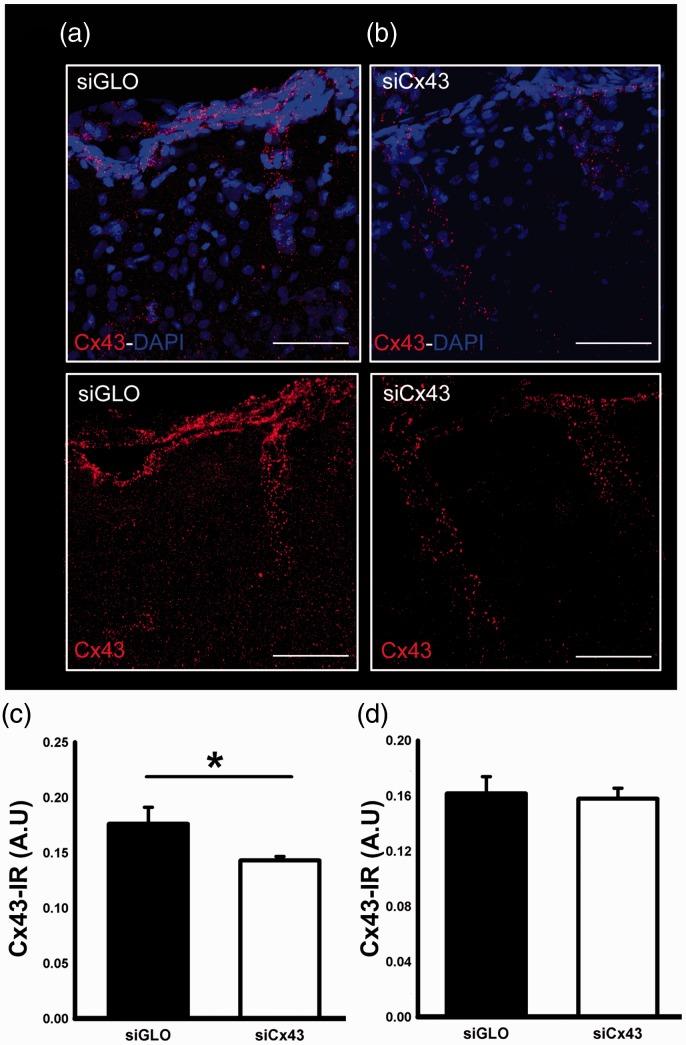
(A, B) Confocal images of Cx43 immunofluorescence staining in the perilesional cortex after siGLO (A) and siCx43 injection (B). (C) Cx43 immunoreactivity was significantly decreased after siCx43 compared with siGLO-treated rats in the perilesional cortex (*p < .05). (D) The expression of Cx43 in the ipsilateral hippocampus was not changed after siCx43 injection. Scale bar = 40 μm. Cx43 = connexin 43; DAPI = 4’,6-diamidino-2-phenylindole; IR = infrared; siCx43 = small interference RNA against Cx43.

In our results, we found that in the perilesional cortex of jTBI animals, there was an initial decrease in Cx43 protein expression followed by a sustained increase at long term (Figure 2). This is in contrast to the Cx30 expression results where only an initial increase in protein expression in the perilesional cortex was observed (Supplementary Figure 1). Interestingly, the pattern of Cx43 changes in the first week is similar to the one described for AQP4 (Fukuda et al., 2012), and our previous study using the same jTBI model and siAQP4 showed beneficial effect of the treatment on the outcome after injury (Fukuda et al., 2013). We therefore decided to study the functional consequences of decreasing Cx43 expression using the siRNA approach after jTBI on the edema formation, astrogliosis, neuronal cell death, and functional recovery. For this purpose, siCx43 was injected in the lesion site after injury as previously described for siAQP4 (Fukuda et al., 2013; Jullienne et al., 2018). Protein changes were measured using IR immunohistochemistry at 3 days post-jTBI to evaluate whether siCx43 was effective in decreasing Cx43 expression. siCx43 induced a significantly lower level of Cx43 expression compared with the siGLO control group at 3 days after the injury (19% decrease) in the perilesional cortex (Figure 3(A) to (C)), but no significant difference was observed in the ipsilateral hippocampus (Figure 3(D)) in accordance with previous reports (Jullienne et al., 2018).

### siCx43 Injection Resulted in Improved Behavioral Outcomes With No Effect on Edema After jTBI

**Figure 4.**
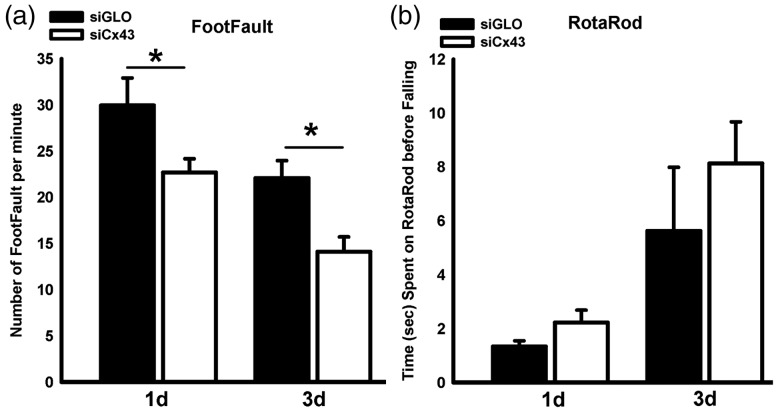
(A) siCx43-treated pups had better functional outcomes as revealed by the foot-fault test. siCx43 group had significantly fewer foot faults than siGLO-treated animals at 1 day (24%) and 33 days (36%) after jTBI (*p < .05). (B) However, siCx43 animals’ performance on the rotarod did not significantly differ with that of siGLO at either 1 day or 3 days. siCx43 = small interference RNA against Cx43.

siCx43 treatment had beneficial effects on the motor behavior after jTBI. The siCx43-treated jTBI group had fewer foot-faults than the control siGLO-treated pups at 1 day and 3 days after injury—24% and 36% respectively, *F*(1, 10)=9.48, *p* < .01 (Figure 4(A)). There was a time effect with a significant decrease of foot-faults between 1 day and 3 days, *F*(1, 10)=26.08, *p* < .0005. siCx43 animals remained on the rotarod longer than siGLO control animals, but this difference was not statistically significant, *F*(1, 10)= 1.38, *p* = .26 (Figure 4(B)). However, there was a significant increase of time spent on rotarod from 1 day to 3 days, *F*(1, 10)=15.26, *p* < .003. Importantly, siCx43 injection in sham mice did not have any effect on the motor behavior for either foot faults (Supplementary Figure 2(A)) or time spent on the rotarod (Supplementary Figure 2(B)) compared with siGLO-treated sham animals.

**Figure 5.**
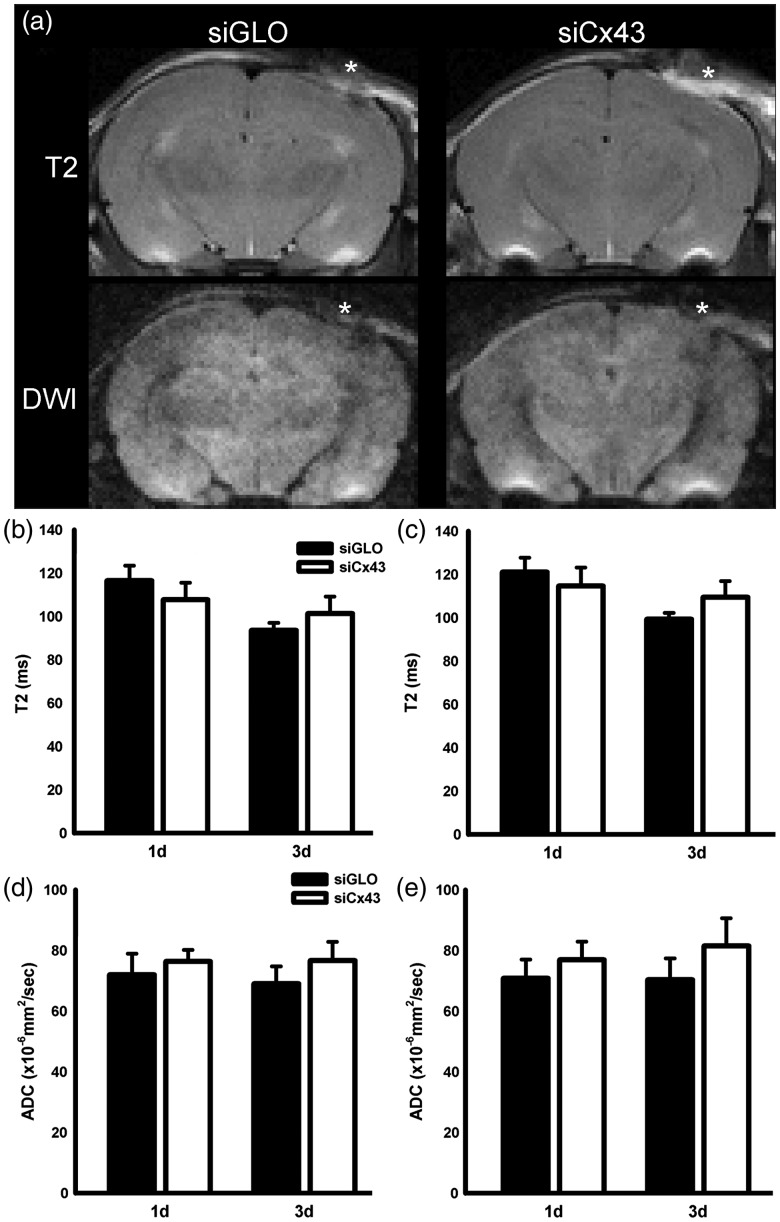
(A) T2-weighted and diffusion-weighted imaging (DWI) of siGLO and siCx43 rats revealed no overt visual differences between the groups. The T2 and DW representative images for each treatment group are from the same rat. The DW image is the weighted image (*b* = 1000) and no diffusion differences are apparent between the two groups (*=TBI site). The extent of edema formation was assessed via MRI using T2 (water content) and ADC (water mobility). T2 values were not significantly different between siCx43 and siGLO pups within the (B) perilesional cortex or (C) ipsilateral hippocampus at either 1 day or 3 days. The ADC value did not significantly differ between siCx43 and siGLO pups within the (D) perilesional cortex or (E) ipsilateral hippocampus at 1 day or 3 days. siCx43 = small interference RNA against Cx43; DWI = diffusion-weighted imaging; ADC = apparent diffusion coefficient.

**Figure 6.**
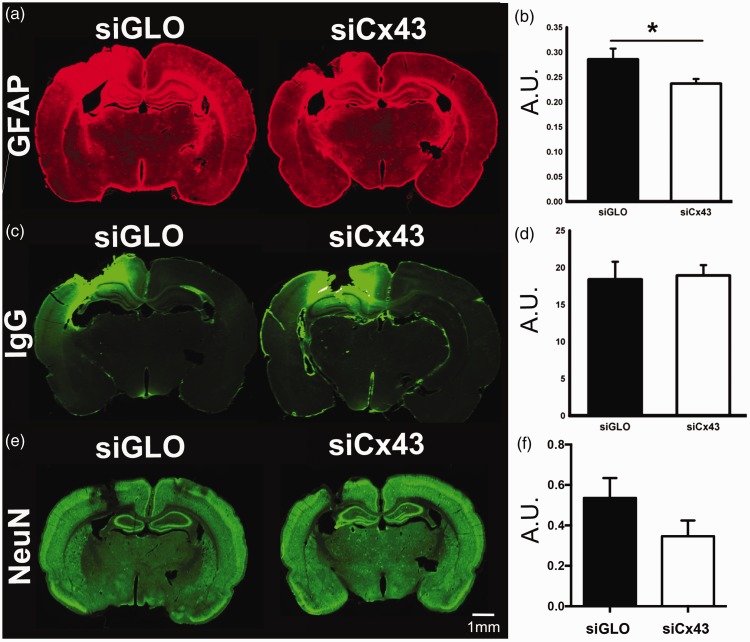
(A) GFAP immunofluorescence. (B) GFAP quantification in the perilesional cortex showed significant decreases in siCx43 compared with siGLO animals. (C) The extent of IgG extravasation (area of extravasation in the cortex divided by the total brain area) was not significantly different between siGLO and siCx43 animals (D), signifying no differences in BBB disruption/permeability (*p < .05). (E) NeuN immunofluorescence as well as quantification within the perilesional cortex showed no differences between siGLO- and siCx43-treated animals (F). Scale bar = 1 mm (*p < .05). siCx43 = small interference RNA against Cx43; GFAP = glial fibrillary acidic protein; IgG = immunoglobulin G; NeuN = neuronal nuclei.

MRI was used to obtain T2 and ADC values in the perilesional cortex and ipsilateral hippocampus at 1 day and 3 days in both siCx43 and siGLO groups (*n* = 5 siGLO and *n* = 7 siCx43 animals). There were no significant differences in T2 values between the siGLO and the siCx43 group (siGLO [1 day: 116.58 ± 6.82 ms; 3 days: 93.54 ± 3.47 ms] and siCx43 [1 day: 107.75 ± 7.84 ms; 3 days: 101.26 ± 7.85 ms]) in the perilesional cortex, *F*(1, 0)=0.003, *p* = .96 (Figure 5(A)). There was time effect with decreased edema at 3 days, *F*(1, 0)= 12.24, *p* = .01. Similarly, no significant differences were observed between siGLO (1 day: 121.53 ± 7.45 ms; 3 days: 99.29 ± 3.01 ms) and siCx43 (1 day: 114.66 ± 8.47 ms; 3 days: 109.45 ± 7.45 ms) in the ipsilateral hippocampus, *F*(1, 0)= 0.04, *p* = .85 (Figure 6(B)). There was time effect also in the ipsilateral hippocampus with decreased edema at 3 days, *F*(1, 0)= 10.55, *p* = .01. There was no interaction of time and treatment for the T2 values in the perilesional cortex or the ipsilateral hippocampus.

ADC values also did not exhibit significant differences between the siGLO and the siCx43 group in the perilesional cortex, *F*(1, 0)=1.29, *p* = .28 (Figure 5(C)). Similarly, no significant difference was observed in the ipsilateral hippocampus between siGLO and siCx43, *F*(1, 0)=0.93, *p* = .36 (Figure 5(D)). There was no time effect or significant interaction of the time and treatment for the ADC values at any of the time points.

### siCx43 Injection Alters GFAP Expression With No Direct Neuroprotective Effect

Because we did not observe any changes in the edema formation between the siCx43- and siGLO-treated animals, AQP4 expression in the perilesional cortex at 3 days post-jTBI was examined (Supplementary Figure 3). AQP4 expression was not different (siGLO: 1719 ± 151.0 A.U., siCx43: 1715 ± 124.0 A.U.) between the groups.

Previously, it has been shown that GFAP immunoreactivity was decreased after ischemia in Cx43 knockout mice and after blocking Cx43 in a TBI model (Nakase et al., 2003; Wu et al., 2013). We investigated in our model of jTBI the effects of siCx43 on astrogliosis after jTBI. In non-jTBI animals, siCx43 injection had no effect on the GFAP staining compared with the siGLO-injected rats (Jullienne et al., 2018). However, we found a 17% decrease in GFAP immunofluorescence in the perilesional cortex of siCx43-treated rat pups compared with siGLO controls (*p* < .05, Figure 6(A) and (B)).

Because astroglial connexins have been hypothesized to play a role in BBB permeability (Ezan et al., 2012), we investigated BBB disruption using IgG immunohistochemistry. At 3 days, there were no significant differences in the extent of IgG extravasation between siCx43 (18.92%) and siGLO controls (18.40% Figure 6(C) and (D)). Thus, there were no differences in the extent of BBB permeability after jTBI between siCx43-treated and siGLO animals.

Finally, we investigated the effects of siCx43 on neuronal survival. NeuN immunoreactivity in the perilesional cortex was not altered between groups (*p* > .05, Figure 6(E) and (F)). Overall, the benefits observed on the motor functions were not supported by decreased neuronal cell death but a change in the astrocytic phenotype.

## Discussion

This is the first report to characterize the modifications in Cx43 protein expression after jTBI and the first to assess the effect of siCx43 on edema and astrogliosis in jTBI. Our novel findings revealed that Cx43 protein levels were increased in the perilesional cortex and ipsilateral CA1 after jTBI. GFAP, as a measure of astrogliosis after jTBI, was also increased up to 60 days after jTBI. Treatment with siCx43 injection after jTBI resulted in improved behavioral outcomes and decreased astrogliosis that were associated with decreased Cx43 protein levels at 3 days postinjury. Therefore, the motor function improvement was not related to neuronal survival but rather to the extent of astrogliosis.

### Changes Seen in Astrocytic Connexins After jTBI: Relation to Edema?

The term *astrocyte network* (Giaume et al., 2010) is frequently used to describe the organization of astrocytes in which individual astrocytes are interconnected through gap junctions composed of connexin proteins: predominantly Cx43 and also Cx30, which facilitate intercellular communication (Seifert et al., 2006; Giaume et al., 2010). Astrocytes and their networks are essential for normal brain function, and disruption of this network by TBI leads to pathological cascades such as excitotoxicity, apoptosis, neuroinflammation, and edema (Sofroniew and Vinters, 2010; Fukuda and Badaut, 2012).

In the brain, the main astrocytic gap junction forming proteins are Cx43 and Cx30 (Nagy et al., 2004). Cx43 and Cx30 stainings have been reported in perivascular astrocyte endfeet (Figure 2(A), Supplementary Figure 1; Jullienne et al., 2018). Although Cx43 and Cx30 are both the main astrocytic connexin proteins, especially in perivascular endfeet, the pattern of change was different between the two proteins, where Cx30 was increased acutely after injury (Supplementary Figure 1(A) and (B)), but Cx43 was decreased initially but then upregulated chronically. Cx43 expression after jTBI was similar to the expression of astrocytic AQP4 in this jTBI model (Fukuda et al., 2012), and for that reason, we hypothesized that Cx43 and AQP4 might have a common role in the process of post-traumatic edema. Interestingly, Cx43 expression was not changed during the edema formation phase at 1 day after jTBI, but both AQP4 and Cx43 were increased during the peak edema phase (3 days) and during the edema resolution phase (7 days; Fukuda et al., 2012). Thus, acutely, the expression profile of Cx43 follows that of AQP4 in support of recent work showing that decreased AQP4 is followed by a decrease in Cx43 expression after silencing AQP4 (siRNA; Jullienne et al., 2018). However, we observed that Cx43 was increased even at 60 days after jTBI, whereas AQP4 was not. This may signify that AQP4 and Cx43 may have a common functional role during the acute period after injury but diverge in expression levels after jTBI. Accordingly, both *in vivo* and in astrocyte cell cultures, AQP4 downregulation led to decreased expression of Cx43 (Jullienne et al., 2018), and in a transgenic mice lacking Cx43 and Cx30, decreased expression of AQP4 was also observed (Ezan et al., 2012). Thus, Cx43 follows the pattern of AQP4 and may contribute to edema resolution at 7 days after injury as speculated previously (Fukuda et al., 2012).

Increased connexin expression after injury has been proposed to be detrimental in several studies of different brain pathologies (Chew et al., 2010), but others have speculated them to be beneficial with no universal consensus (Farahani et al., 2005; Wallraff et al., 2006; Zweckberger and Plesnila, 2009). In an adult model of TBI using a lateral fluid percussion injury in adult rats, a similar pattern of Cx43 immunoreactivity was reported as in our model of jTBI, wherein an initial reduction was followed by increases in the hippocampus and the cortex (Ohsumi et al., 2006). However, it is interesting to note that the acute reduction was observed at 6 hours and then increased at 24 hours (Ohsumi et al., 2006). This apparent shift in the Cx43 time course may be due to different injury models or different age, highlighting the importance of treating jTBI as a different pathology than adult TBI (Giza et al., 2007). Thus, it is likely that the determining factor of whether connexin under- or overexpression is beneficial or detrimental is determined by the injury type and the time point after injury, similar to what has been hypothesized for AQP4 where inhibition of AQP4 would be beneficial during the edema formation phase but not during the edema resolution phase (Fukuda et al., 2012, 2013).

We hypothesized that astrocytes most likely play a multifaceted role in the edema process. Initially, AQP4 permits water entry into the brain across the BBB. Second, the astrocyte network of gap junction channels allows the spread of water accumulation from the primary injured astrocytes to surrounding glia, causing them to swell as well (Figure 1(A)). Furthermore, non-gap junction forming hemichannels may have an additional effect in the post-injury cascade via excitotoxicity through extracellular ATP and glutamate signaling (Bennett et al., 2012; Kar et al., 2012). Because the changes in Cx43 expression after jTBI followed AQP4 expression changes, we hypothesized that Cx43 is the main gap junction protein involved in the spread of edema. We tested this hypothesis to examine if decreasing Cx43 after jTBI would result in decreased edema, leading to improved recovery using siCx43.

### The Effect of siCx43 Injection After jTBI

Injection of siCx43 resulted in improved motor function after jTBI with siCx43-treated animals having fewer foot faults than siGLO-treated animals (Figure 4(A)). However, even though siCx43 animals spent more time on the rotarod than siGLO animals, this difference was not significant (Figure 4(B)). We cannot explain the reason for this discrepancy between the motor function tests, but our results are in line with previous studies where the foot-fault test had better discrimination between groups compared with the rotarod test (Ajao et al., 2012; Kamper et al., 2013). We observed a decrease in GFAP immunoreactivity after siCx43 injection after jTBI (Figure 6). This decrease in GFAP is in accordance with an adult TBI study using antisense oligonucleotide pretreatment against Cx43 (Wu et al., 2013) and another study in which gap junction inhibitors, carbenoxolone and octanol, were administered in adult rats that underwent a stab wound to mimic brain injury (Andersson et al., 2011). Although the exact function and implication of changes in GFAP immunoreactivity is debated, it is commonly regarded to be associated with astrogliosis (Sofroniew, 2005; Sofroniew and Vinters, 2010). Some evidence suggests that reactive astrogliosis may contribute to worsened secondary injury depending on the time point and injury model (Laird et al., 2008).

To study edema after jTBI, we used MRI. DWI and T2WI imaging are routinely used clinically as a measure of edema (Galloway et al., 2008; Chastain et al., 2009) and in animal models, including juvenile animals (Badaut et al., 2011; Fukuda et al., 2012, 2013). We used these clinically relevant imaging modalities to map the time course of evolution of edema. ADC is a DWI parameter that measures water mobility, and T2 is a measure of water content. In a previous study using siAQP4, both ADC and T2 were decreased in jTBI animals injected with siAQP4, signifying decreased edema, which was associated with improved behavioral outcomes and decreased reactive astrogliosis (Fukuda et al., 2013). Thus, we hypothesized that the inhibition of edema and suppression of secondary injury spread by limiting water diffusion through the astrocyte network could be achieved by either blocking water channels (AQP4, astrocyte-BBB) or gap junctions (connexins, astrocyte–astrocyte communication). However, contrary to our initial hypothesis, postinjury administration of siCx43 did not result in a significant decrease in edema or BBB disruption (Figure 6).

There are several possible explanations for these observations. First, it is possible that both Cx43 and Cx30 must be knocked down to decrease edema. Indeed, it is very plausible that water may still be propagated across the astrocyte network from the primary injury site to secondary injury sites through Cx30, compensating for the downregulated Cx43 channels. It would be interesting to see the effect of double knockdown treatment for Cx43/Cx30 in future studies; however, the siRNA approach is not the best to achieve this because it is very challenging to have a double knockdown in the same cells. However, it is also important to note that combination therapies do not always have benefit over monotherapies. In fact, a recent review showed that out of six combination treatments for TBI, only two showed modest improvements over monotherapy, with some showing decrease of efficacy over monotreatments (Margulies et al., 2016). Second, pretreatment/preconditioning with a gap junction inhibitor found a greater difference between the control group and the treated group than posttreatment (Perez Velazquez et al., 2006; Andersson et al., 2011). This antisense-oligodeoxynucleotide against Cx43 treatment may have resulted in decreased edema due to the pre-injury injection of the drug. Third, Cx43 in juvenile animals may not be a key player in the edema process. Water propagation or clearance could be a function that is more central to other astrocytic proteins, namely AQP4 (Badaut et al., 2011). Recent results showed that the injection of siCx43 does not modify ADC in contrast to injection of siAQP4 (Jullienne et al., 2018). Altogether, these results continue to support the hypothesis that AQP4 is the main contributor in edema formation/resolution after jTBI. However, it is possible that variations in the alternates suggested earlier may also contribute. Indeed, astrocytic Cx43 and Cx30 conditional double knockout mice have a leaky BBB and astrocyte endfeet swelling (Ezan et al., 2012). Further, in a sheep model of global ischemia, Cx43 inhibition through a specific mimetic peptide was shown to result in increased neuronal and oligodendrocyte cell count if the peptide was given after the ischemia but not before and during ischemia (Davidson et al., 2013).

Although TBI studies on astrocytic Cx43 are sparse, increased Cx43 has been associated with other brain pathologies (Chew et al., 2010). Ischemic stroke studies have reported beneficial results by specific inhibition of Cx43 in *in vitro* models (Chew et al., 2010) and general gap junction inhibition *in vivo* (Perez Velazquez et al., 2006; Andersson et al., 2011). Thus, siCx43 may be a unique and useful new technical approach to study *in vivo* the involvement of Cx43 as well as a potential therapeutic tool in other brain injury models as well. However, it is interesting to note that in models of cerebral hemorrhage, general gap junction inhibitors such as carbenoxolone and octanol have elicited detrimental effects, more specifically in a model of intracerebral hemorrhage (Manaenko et al., 2009) and experimental subarachnoid hemorrhage (Ayer et al., 2010). As proposed by these authors, brain hemorrhage may follow a different path than TBI and stroke—namely that the injurious factors are extracellular, and the intra-astrocellular bridges formed by gap junctions may not be as important (Ayer et al., 2010).

### Limitations of the Study

Our current work has several limitations: (a) We previously observed significant improvement using siAQP4 cortical injections after jTBI with improvement in motor function at 3 days, decreased BBB permeability, astrogliosis, and neuronal cell death (Fukuda et al., 2013), even with approximately 25% decrease in AQP4 protein expression (Badaut et al., 2011; Fukuda et al., 2013). The degree of Cx43 expression was similar with a 19% decrease after siCx43 injection, but the benefits of the treatment were limited relative to siAQP4 treatment. However, we cannot exclude the possibility that larger decreases in Cx43 expression might have an effect on edema formation and change the outcome after treatment, which would be interesting to address in future studies. (b) We focused only on targeting Cx43 in the present study based on our previous studies examining the effects of siAQP4 on Cx43 expression (Jullienne et al., 2018). However, the role of Cx30 in edema and astrogliosis after jTBI cannot be ruled out.

## Conclusion

In conclusion, we show here for the first time the temporal changes in Cx43 and GFAP expression after jTBI. We also show that siCx43 injection after injury in juvenile animals results in improved sensorimotor behavioral recovery, associated with decreased Cx43 and reactive astrogliosis but is not associated with changes in edema formation. Future studies could further examine the mechanistic pathways underlying the beneficiary effects such as decreased cell death, neuroinflammation, or decreased excitotoxicity.

## Summary

Blocking the temporal changes in Cx43 expression using siRNA improves sensorimotor recovery associated with decrease in reactive astrocytes after jTBI.

## Supplemental Material

Supplemental material for Small Interference RNA Targeting Connexin-43 Improves Motor Function and Limits Astrogliosis After Juvenile Traumatic Brain Injury Click here for additional data file. Supplemental Material for Small Interference RNA Targeting Connexin-43 Improves Motor Function and Limits Astrogliosis After Juvenile Traumatic Brain Injury by Aleksandra Ichkova, Andrew M. Fukuda, Nina Nishiyama, Germaine Paris, Andre Obenaus and Jerome Badaut in ASN Neuro

## References

[bibr1-1759091419847090] Ajao D. O. Pop V. Kamper J. E. Adami A. Rudobeck E. Huang L. Vlkolinsky R. Hartman R. E. Ashwal S. Obenaus A. Badaut J. (2012). Traumatic brain injury in young rats leads to progressive behavioral deficits coincident with altered tissue properties in adulthood. J Neurotrauma, 29, 2060–2074. 22697253 10.1089/neu.2011.1883 PMC3408248

[bibr2-1759091419847090] Andersson H. C. Anderson M. F. Porritt M. J. Nodin C. Blomstrand F. Nilsson M. (2011). Trauma-induced reactive gliosis is reduced after treatment with octanol and carbenoxolone. Neurol Res, 33, 614–624. 21708071 10.1179/1743132810Y.0000000020

[bibr3-1759091419847090] Andrade-Rozental A. F. Rozental R. Hopperstad M. G. Wu J. K. Vrionis F. D. Spray D. C. (2000). Gap junctions: The “kiss of death” and the “kiss of life”. Brain Res Brain Res Rev, 32, 308–315. 10751679 10.1016/s0165-0173(99)00099-5

[bibr4-1759091419847090] Ayer R. Chen W. Sugawara T. Suzuki H. Zhang J. H. (2010). Role of gap junctions in early brain injury following subarachnoid hemorrhage. Brain Res, 1315, 150–158. 20018179 10.1016/j.brainres.2009.12.016 PMC2844087

[bibr5-1759091419847090] Badaut J. Ashwal S. Adami A. Tone B. Recker R. Spagnoli D. Ternon B. Obenaus A. (2011). Brain water mobility decreases after astrocytic aquaporin-4 inhibition using RNA interference. J Cereb Blood Flow Metab, 31, 819–831. 20877385 10.1038/jcbfm.2010.163 PMC3063618

[bibr6-1759091419847090] Bennett M. V. Garre J. M. Orellana J. A. Bukauskas F. F. Nedergaard M. Saez J. C. (2012). Connexin and pannexin hemichannels in inflammatory responses of glia and neurons. Brain Res, 1487, 3–15. 22975435 10.1016/j.brainres.2012.08.042 PMC3627726

[bibr7-1759091419847090] Binder D. K. Oshio K. Ma T. Verkman A. S. Manley G. T. (2004). Increased seizure threshold in mice lacking aquaporin-4 water channels. Neuroreport, 15, 259–262. 15076748 10.1097/00001756-200402090-00009

[bibr8-1759091419847090] Binder D. K. Yao X. Zador Z. Sick T. J. Verkman A. S. Manley G. T. (2006). Increased seizure duration and slowed potassium kinetics in mice lacking aquaporin-4 water channels. Glia, 53, 631–636. 16470808 10.1002/glia.20318

[bibr9-1759091419847090] Chastain C. A. Oyoyo U. E. Zipperman M. Joo E. Ashwal S. Shutter L. A. Tong K. A. (2009). Predicting outcomes of traumatic brain injury by imaging modality and injury distribution. J Neurotrauma, 26, 1183–1196. 19317591 10.1089/neu.2008.0650

[bibr10-1759091419847090] Chew S. S. Johnson C. S. Green C. R. Danesh-Meyer H. V. (2010). Role of connexin43 in central nervous system injury. Exp Neurol, 225, 250–261. 20655909 10.1016/j.expneurol.2010.07.014

[bibr11-1759091419847090] Clement T. Rodriguez-Grande B. Badaut J. (2018). Aquaporins in brain edema. *J Neurosci Res*, 2015. doi: 10.1002/jnr.24354. 10.1002/jnr.24354 30430614

[bibr12-1759091419847090] Davidson J. O. Green C. R. Nicholson L. F. Bennet L. Gunn A. J. (2013). Connexin hemichannel blockade is neuroprotective after, but not during, global cerebral ischemia in near-term fetal sheep. Exp Neurol, 248, 301–308. 23838537 10.1016/j.expneurol.2013.06.026

[bibr13-1759091419847090] Donkin J. J. Vink R. (2010). Mechanisms of cerebral edema in traumatic brain injury: Therapeutic developments. Curr Opin Neurol, 23, 293–299. 20168229 10.1097/WCO.0b013e328337f451

[bibr14-1759091419847090] Ezan P. Andre P. Cisternino S. Saubamea B. Boulay A. C. Doutremer S. Thomas M. A. Quenech'du N. Giaume C. Cohen-Salmon M. (2012). Deletion of astroglial connexins weakens the blood-brain barrier. J Cereb Blood Flow Metab, 32, 1457–1467. 22472609 10.1038/jcbfm.2012.45 PMC3421093

[bibr15-1759091419847090] Farahani R. Pina-Benabou M. H. Kyrozis A. Siddiq A. Barradas P. C. Chiu F. C. Cavalcante L. A. Lai J. C. Stanton P. K. Rozental R. (2005). Alterations in metabolism and gap junction expression may determine the role of astrocytes as “good samaritans” or executioners. Glia, 50, 351–361. 15846800 10.1002/glia.20213

[bibr16-1759091419847090] Faul M. Xu L. Wald M. M. Coronado V. (2010). Traumatic brain injury in the United States: Emergency department visits, hospitalizations, and deaths, 2002–2006. Atlanta, GA: National Center for Injury Prevention and Control, CDC.

[bibr17-1759091419847090] Fukuda A. M. Adami A. Pop V. Bellone J. A. Coats J. S. Hartman R. E. Ashwal S. Obenaus A. Badaut J. (2013). Posttraumatic reduction of edema with aquaporin-4 RNA interference improves acute and chronic functional recovery. J Cereb Blood Flow Metab, 33, 1621–1632. 23899928 10.1038/jcbfm.2013.118 PMC3790933

[bibr18-1759091419847090] Fukuda A. M. Badaut J. (2012). Aquaporin 4: A player in cerebral edema and neuroinflammation. J Neuroinflammation, 9, 279. 23270503 10.1186/1742-2094-9-279 PMC3552817

[bibr19-1759091419847090] Fukuda A. M. Pop V. Spagnoli D. Ashwal S. Obenaus A. Badaut J. (2012). Delayed increase of astrocytic aquaporin 4 after juvenile traumatic brain injury: Possible role in edema resolution? Neuroscience, 222, 366–378. 22728101 10.1016/j.neuroscience.2012.06.033 PMC3482829

[bibr20-1759091419847090] Galloway N. R. Tong K. A. Ashwal S. Oyoyo U. Obenaus A. (2008). Diffusion-weighted imaging improves outcome prediction in pediatric traumatic brain injury. J Neurotrauma, 25, 1153–1162. 18842104 10.1089/neu.2007.0494

[bibr21-1759091419847090] Giaume C. Koulakoff A. Roux L. Holcman D. Rouach N. (2010). Astroglial networks: A step further in neuroglial and gliovascular interactions. Nat Rev Neurosci, 11, 87–99. 20087359 10.1038/nrn2757

[bibr22-1759091419847090] Giaume C. McCarthy K. D. (1996). Control of gap-junctional communication in astrocytic networks. Trends Neurosci, 19, 319–325. 8843600 10.1016/0166-2236(96)10046-1

[bibr23-1759091419847090] Giza C. C. Mink R. B. Madikians A. (2007). Pediatric traumatic brain injury: Not just little adults. Curr Opin Crit Care, 13, 143–152. 17327734 10.1097/MCC.0b013e32808255dc

[bibr24-1759091419847090] Grange-Messent V. Raison D. Bouchaud C. (1996). Compared effects of extracellular K+ ions and soman, a neurotoxic, on cerebral astrocyte morphology. An in vitro study. J Submicrosc Cytol Pathol, 28, 151–159. 8964039

[bibr25-1759091419847090] Herve J. C. Derangeon M. (2013). Gap-junction-mediated cell-to-cell communication. Cell Tissue Res, 352, 21–31. 22940728 10.1007/s00441-012-1485-6

[bibr26-1759091419847090] Hirt L. Ternon B. Price M. Mastour N. Brunet J. F. Badaut J. (2009). Protective role of early aquaporin 4 induction against postischemic edema formation. J Cereb Blood Flow Metab, 29, 423–433. 18985050 10.1038/jcbfm.2008.133

[bibr27-1759091419847090] Jullienne A. Fukuda A. M. Ichkova A. Nishiyama N. Aussudre J. Obenaus A. Badaut J. (2018). Modulating the water channel AQP4 alters miRNA expression, astrocyte connectivity and water diffusion in the rodent brain. Sci Rep, 8, 4186. 29520011 10.1038/s41598-018-22268-y PMC5843607

[bibr50-1759091419847090] Kamper, J. E., Pop, V., Fukuda, A. M., Ajao, D. O., Hartman, R. E., & Badaut, J. (2013). Juvenile traumatic brain injury evolves into a chronic brain disorder: Behavioral and histological changes over 6months. *Exp Neurol*, *250*, 8–19. 10.1016/j.expneurol.2013.09.016 PMC3895624 24076005

[bibr28-1759091419847090] Kar R. Batra N. Riquelme M. A. Jiang J. X. (2012). Biological role of connexin intercellular channels and hemichannels. Arch Biochem Biophys, 524, 2–15. 22430362 10.1016/j.abb.2012.03.008 PMC3376239

[bibr29-1759091419847090] Laird M. D. Vender J. R. Dhandapani K. M. (2008). Opposing roles for reactive astrocytes following traumatic brain injury. Neurosignals, 16, 154–164. 18253055 10.1159/000111560

[bibr30-1759091419847090] Manaenko A. Lekic T. Sozen T. Tsuchiyama R. Zhang J. H. Tang J. (2009). Effect of gap junction inhibition on intracerebral hemorrhage-induced brain injury in mice. Neurol Res, 31, 173–178. 19298758 10.1179/174313209X393591 PMC6866672

[bibr31-1759091419847090] Morales D. M. Marklund N. Lebold D. Thompson H. J. Pitkanen A. Maxwell W. L. Longhi L. Laurer H. Maegele M. Neugebauer E. Graham D. I. Stocchetti N. McIntosh T. K. (2005). Experimental models of traumatic brain injury: Do we really need to build a better mousetrap? Neuroscience, 136, 971–989. 16242846 10.1016/j.neuroscience.2005.08.030

[bibr51-1759091419847090] Margulies, S., Anderson, G., Atif, F., Badaut, J., Clark, R., Empey, P., Guseva, M., Hoane, M., Huh, J., Pauly, J., Raghupathi, R., Scheff, S., Stein, D., Tang, H., & Hicks, M. (2016). Combination Therapies for Traumatic Brain Injury: Retrospective Considerations. *J Neurotrauma*, *33*, 101–112. 10.1089/neu.2014.3855 PMC4700397 25970337

[bibr32-1759091419847090] Nagy J. I. Dudek F. E. Rash J. E. (2004). Update on connexins and gap junctions in neurons and glia in the mammalian nervous system. Brain Res Brain Res Rev, 47, 191–215. 15572172 10.1016/j.brainresrev.2004.05.005

[bibr33-1759091419847090] Nakase T. Fushiki S. Naus C. C. (2003). Astrocytic gap junctions composed of connexin 43 reduce apoptotic neuronal damage in cerebral ischemia. Stroke, 34, 1987–1993. 12843358 10.1161/01.STR.0000079814.72027.34

[bibr34-1759091419847090] Niermann H. Amiry-Moghaddam M. Holthoff K. Witte O. W. Ottersen O. P. (2001). A novel role of vasopressin in the brain: Modulation of activity-dependent water flux in the neocortex. J Neurosci, 21, 3045–3051. 11312289 10.1523/JNEUROSCI.21-09-03045.2001 PMC6762582

[bibr35-1759091419847090] Ohsumi A. Nawashiro H. Otani N. Ooigawa H. Toyooka T. Yano A. Nomura N. Shima K. (2006). Alteration of gap junction proteins (connexins) following lateral fluid percussion injury in rats. Acta Neurochir Suppl, 96, 148–150. 16671444 10.1007/3-211-30714-1_33

[bibr36-1759091419847090] Perez Velazquez J. L. Frantseva M. V. Naus C. C. (2003). Gap junctions and neuronal injury: Protectants or executioners? Neuroscientist, 9, 5–9. 12580335 10.1177/1073858402239586

[bibr37-1759091419847090] Perez Velazquez J. L. Kokarovtseva L. Sarbaziha R. Jeyapalan Z. Leshchenko Y. (2006). Role of gap junctional coupling in astrocytic networks in the determination of global ischaemia-induced oxidative stress and hippocampal damage. Eur J Neurosci, 23, 1–10. 16420410 10.1111/j.1460-9568.2005.04523.x

[bibr38-1759091419847090] Plesnila N. (2016). The immune system in traumatic brain injury. Curr Opin Pharmacol, 26, 110–117. 26613129 10.1016/j.coph.2015.10.008

[bibr39-1759091419847090] Pop V. Badaut J. (2011). A neurovascular perspective for long-term changes after brain trauma. Transl Stroke Res, 2, 533–545. 22350620 10.1007/s12975-011-0126-9 PMC3281750

[bibr40-1759091419847090] Rash J. E. Yasumura T. Davidson K. G. Furman C. S. Dudek F. E. Nagy J. I. (2001). Identification of cells expressing Cx43, Cx30, Cx26, Cx32 and Cx36 in gap junctions of rat brain and spinal cord. Cell Commun Adhes, 8, 315–320. 12064610 10.3109/15419060109080745 PMC1805789

[bibr41-1759091419847090] Risher W. C. Andrew R. D. Kirov S. A. (2009). Real-time passive volume responses of astrocytes to acute osmotic and ischemic stress in cortical slices and in vivo revealed by two-photon microscopy. Glia, 57, 207–221. 18720409 10.1002/glia.20747 PMC2635108

[bibr42-1759091419847090] Seifert G. Schilling K. Steinhauser C. (2006). Astrocyte dysfunction in neurological disorders: A molecular perspective. Nat Rev Neurosci, 7, 194–206. 16495941 10.1038/nrn1870

[bibr43-1759091419847090] Sofroniew M. V. (2005). Reactive astrocytes in neural repair and protection. Neuroscientist, 11, 400–407. 16151042 10.1177/1073858405278321

[bibr44-1759091419847090] Sofroniew M. V. Vinters H. V. (2010). Astrocytes: Biology and pathology. Acta Neuropathol, 119, 7–35. 20012068 10.1007/s00401-009-0619-8 PMC2799634

[bibr45-1759091419847090] Wallraff A. Kohling R. Heinemann U. Theis M. Willecke K. Steinhauser C. (2006). The impact of astrocytic gap junctional coupling on potassium buffering in the hippocampus. J Neurosci, 26, 5438–5447. 16707796 10.1523/JNEUROSCI.0037-06.2006 PMC6675300

[bibr46-1759091419847090] Wu Z. Xu H. He Y. Yang G. Liao C. Gao W. Liang M. He X. (2013). Antisense oligodeoxynucleotides targeting connexin43 reduce cerebral astrocytosis and edema in a rat model of traumatic brain injury. Neurol Res, 35, 255–262. 23485053 10.1179/1743132813Y.0000000165

[bibr47-1759091419847090] Zweckberger K. Plesnila N. (2009). Anatibant, a selective non-peptide bradykinin B2 receptor antagonist, reduces intracranial hypertension and histopathological damage after experimental traumatic brain injury. Neurosci Lett, 454, 115–117. 19429066 10.1016/j.neulet.2009.02.014

