# Supplementary material for: Small Interference RNA Targeting Connexin-43 Improves Motor Function and Limits Astrogliosis After Juvenile Traumatic Brain Injury
Source: ASN Neuro. 2019 Jun 13;11:1759091419847090. doi: 10.1177/1759091419847090 (PMC6566476; doi:10.1177/1759091419847090)
Supplement: Supplemental material for Small Interference RNA Targeting Connexin-43 Improves Motor Function and Limits Astrogliosis After Juvenile Traumatic Brain Injury [file Supplemental_Material.pdf]

SMALL INTERFERENCE RNA TARGETING CONNEXIN-43 IMPROVES MOTOR FUNCTION AND LIMITS ASTROGLIOSIS AFTER JUVENILE TRAUMATIC BRAIN INJURY

Running head: Role of connexin 43 in jTBI

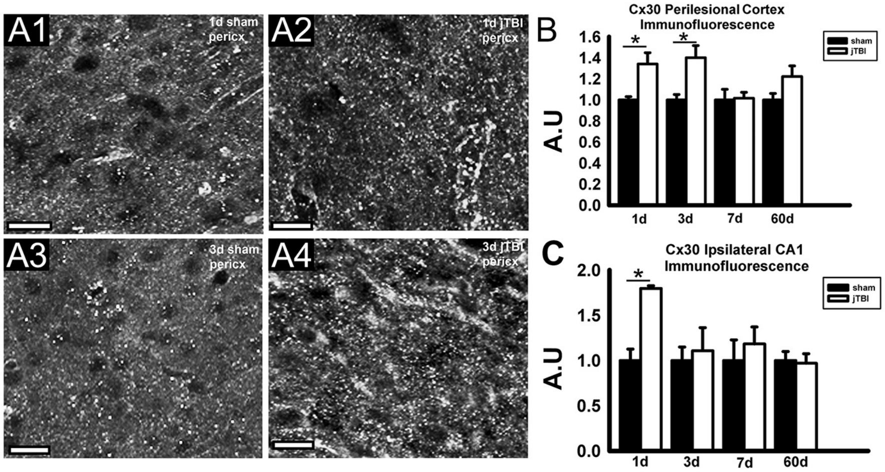

Supplementary figure 1

(A1, A2, A3, A4) Cx30 staining quantification in the perilesional cortex showed increased Cx30 immunoreactivity at 1d (A2) and 3d (A4) after jTBI as compared to sham (A1, A3). (B) Quantification of Cx30 immunoreactivity in the perilesional cortex (*$p<0.05$). (C) Quantification of Cx30 immunoreactivity in the ipsilateral hippocampus showed a significant increase at 1d (*$p<0.05$). scale bar 40 μm

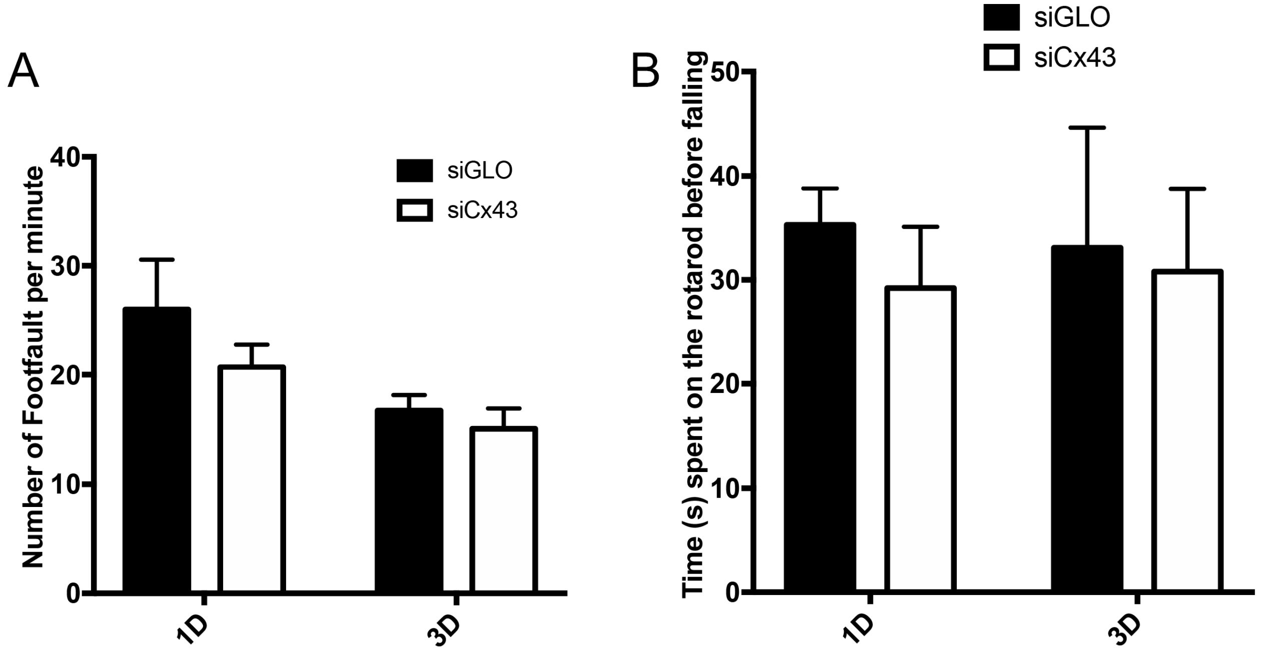

Supplementary figure 2

Supplementary figure 2

A. Injection of siCx43 did not have an effect on the number of foot faults at 1d or 3d after injection, compared to siGLO, (n=10 per groups, p>0.05). B. There was no difference in the time spent on the rotarod between siCx43 and siGLO groups at 3d after injection, (n=10 per groups, p>0.05).

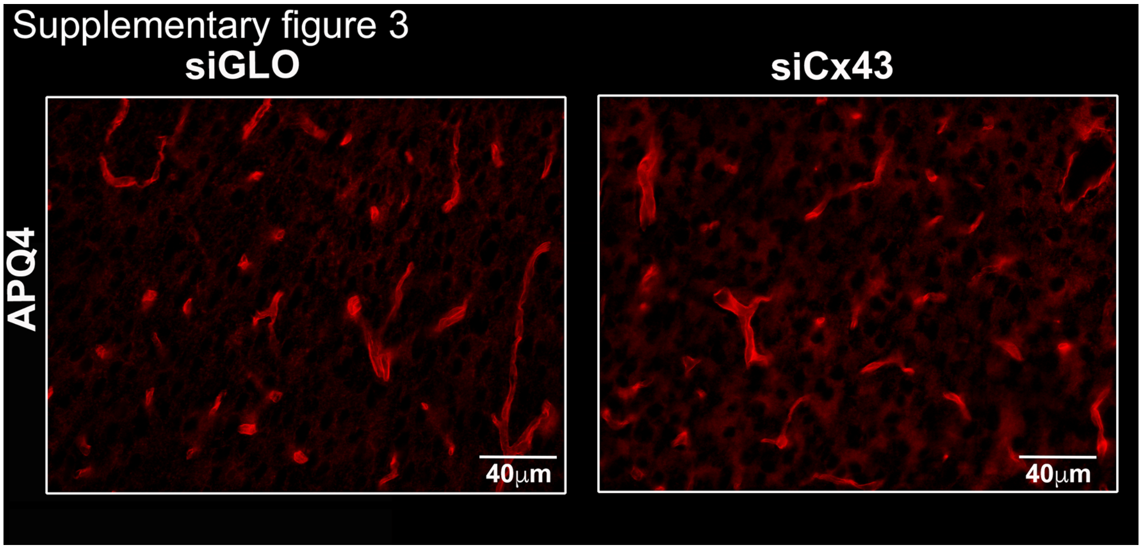

Supplementary figure 3

AQP4 expression in the perilesional cortex was not different in between siGLO and siCx43 treated jTBI animals, scale bar 40 μm
